# MHY498 Nanosuspensions for Improved Topical Drug Delivery: Understanding of Its Solubility Behavior in DEGME + Water Mixtures and Preparation of Nanosuspension Using Box–Behnken Design

**DOI:** 10.3390/pharmaceutics18010127

**Published:** 2026-01-20

**Authors:** Eun-Sol Ha, Ha Nim Lee, Seon-Kwang Lee, Ji-Su Jeong, Jeong-Soo Kim, Hyung Ryong Moon, In-hwan Baek, Heejun Park, Min-Soo Kim

**Affiliations:** 1 College of Pharmacy and Research Institute for Drug Development, Pusan National University, 63 Busandaehak-ro, Geumjeong-gu, Busan 46241, Republic of Korea; edel@pusan.ac.kr (E.-S.H.); gksla64@pusan.ac.kr (H.N.L.); lsk7079@pusan.ac.kr (S.-K.L.); sui15@pusan.ac.kr (J.-S.J.); mhr108@pusan.ac.kr (H.R.M.); 2 Dong-A ST Co., Ltd., Giheung-gu, Yongin 17073, Republic of Korea; js_kim@donga.co.kr; 3 College of Pharmacy, Kyungsung University, 309, Suyeong-ro, Nam-gu, Busan 48434, Republic of Korea; baek@ks.ac.kr; 4 College of Pharmacy, Duksung Women’s University, 33, Samyangro 144-gil, Dobong-gu, Seoul 01369, Republic of Korea

**Keywords:** MHY498, solubility, correlation, antisolvent precipitation, nanosuspension

## Abstract

**Background/Objectives**: MHY498, a tyrosinase inhibitor, exhibits poor water solubility, which limits its topical delivery. Despite the importance of solubility data in rational formulation design, comprehensive information on its solubility behavior in various solvents and across a range of temperatures remains limited. Thus, this study aimed to systematically evaluate the solubility characteristics of MHY498 and to develop a nanosuspension formulation using an antisolvent precipitation approach to facilitate the development of an optimized topical formulation. **Methods**: In this study, we measured the solubility of MHY498 in various monosolvents and diethylene glycol monoethyl ether (DEGME) + water solvent mixtures at 293.15–313.15 K using a solid–liquid equilibrium technique. Based on these solubility data, MHY498 nanosuspensions were prepared via antisolvent precipitation guided by a Box–Behnken design matrix. In vitro skin permeability was also assessed using a Franz diffusion cell system to assess the topical delivery potential of the MHY498 nanosuspensions. **Results**: Among the investigated monosolvents, MHY498 exhibited the highest solubility in dimethylformamide, dimethylacetamide, DEGME, while the lowest solubility was observed in water. The solubility increased with temperature and DEGME content in solvent mixtures, and the experimental data were well described by thermodynamic and semi-empirical models, indicating an endothermic and spontaneous dissolution process. Solvent–solute interaction analysis revealed that hydrogen-bonding and nonspecific polarity interactions played key roles in enhancing MHY498 solubility. All nanosuspensions prepared within the design space exhibited particle sizes below 150 nm, and the optimized formulation achieved an average particle size of 28.1 nm. The optimized nanosuspension demonstrated a 3.3-fold increase in the cumulative permeated amounts compared with the conventional microsuspension. **Conclusions**: These findings demonstrate that a rational solvent selection strategy based on thermodynamic solubility analysis and antisolvent precipitation enables effective nanosuspension formulation of MHY498. The DEGME–water system was identified as a formulation-relevant solvent environment that supports both adequate drug solubilization and reproducible formation of nanosized particles. The resulting nanosuspension exhibited favorable particle size characteristics and enhanced formulation feasibility for topical applications. Therefore, it was shown that the developed nanosuspension system, established through a solubility-driven systematic approach, represents a promising strategy for improving topical delivery of MHY498.

## 1. Introduction

**Figure 1:**
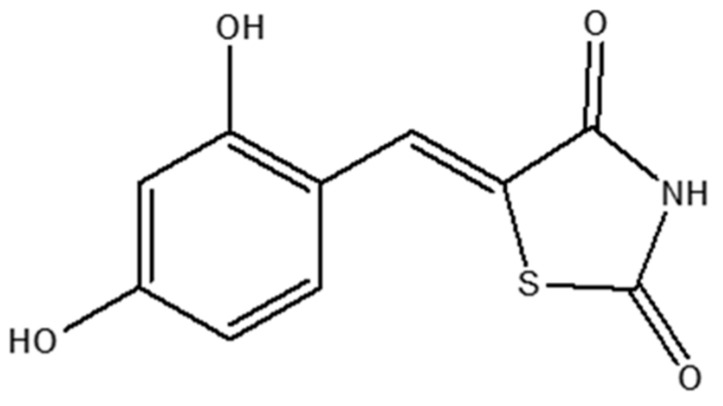
Molecular structure of MHY498.

(Z)-5-(2,4-dihydroxybenzylidene)thiazolidine-2,4-dione (MHY498, CAS # 1208535-06-1) is a tyrosinase inhibitor containing imidazole and 2,4-dihydroxy phenyl moieties, which exists as a yellow crystalline powder (C_10_H_7_NO_4_S) with a molecular weight of 237.23 g∙mol^−1^ (Figure 1). MHY498 inhibits tyrosinase activity in a dose-dependent manner and is more potent than the tyrosinase inhibitor kojic acid. Based on the results of experiments with B16F10, melanoma cells, and α-melanocyte stimulating hormone (α-MSH), MHY498 inhibited the activity of tyrosinase in a dose-dependent manner and reduced melanogenesis without inducing cytotoxicity, whereas kojic acid induced cell toxicity at the same concentration. These findings support the potential of MHY498 as a therapeutic agent for preventing and treating skin pigmentation. MHY498 is listed in the International Nomenclature Cosmetic Ingredient (INCI) catalog as dihydroxybenzylidene thiazolidinedione (ID 28484) and has been incorporated into commercial cosmetic products. Despite its potency, topical delivery is challenging due to low aqueous solubility and the strong barrier function of the stratum corneum [1,2,3,4].

Formulation strategies, including prodrug design, eutectic mixtures, complexation, and lipid-based systems, such as microemulsions, liposomes, and vesicles, have been explored to optimize the properties of active ingredients and vehicles and enhance drug penetration across the stratum corneum [5,6,7,8,9,10,11,12,13,14]. Understanding MHY498 solubility in water or water-solvent mixtures at different temperatures is crucial for optimizing skin penetration, designing solubilized topical formulations (creams, gels, lotions), and developing lipid-based carriers, such as solid–lipid nanoparticles [15,16,17,18]. Additionally, understanding the solubility of MHY498 in various solvents used for preparing and purifying the product is necessary. Nevertheless, to date, the solubility data for MHY498 in various monosolvents or solvent mixtures across temperatures remain lacking. Moreover, formulation studies addressing solubility-driven strategies for MHY498 remain scarce, highlighting the need for a systematic investigation linking solubility to formulation design.

In this study, we measured the mole-fraction solubility of MHY498 in diverse monosolvents and diethylene glycol monoethyl ether (DEGME) + water solvent mixtures using a solid–liquid equilibrium technique. The solubility data for MHY498 were fitted to thermodynamic models, including van’t Hoff, modified Apelblat, simplified CNIBS/R-K, Jouyban–Acree, Ma, Sun, modified Wilson, mixture response surface (MRS), and Yalkowsky-Roseman models. Solvent effects were further analyzed using the Kamlet–Taft linear solvation energy relationship (KAT-LSER) model, and thermodynamic parameters of dissolution were estimated using van’t Hoff analysis. Based on these findings, MHY498 nanosuspensions were prepared by antisolvent precipitation and evaluated in vitro to assess skin permeation, providing insights into effective topical delivery.

## 2. Materials and Methods

### 2.1. Materials

(*Z*)-5-(2,4-dihydroxybenzylidene)thiazolidine-2,4-dione (MHY498, C_10_H_7_NO_4_S), with a molecular weight of 237.23 g∙mol^−1^, was synthesized by Professor Hyung Ryong Moon in the Department of Manufacturing Pharmacy at Pusan National University, Republic of Korea. Acetonitrile, 1-butanol, dimethylformamide (DMF), dimethylacetamide (DMA), dimethyl sulfoxide (DMSO), ethanol, methanol, N-methyl-2-pyrrolidone (NMP), 1-propanol, and 2-propanol were purchased from Honeywell Burdick and Jackson (Morristown, NJ, USA). Acetone, ethyl acetate, and tetrahydrofuran (THF) were purchased from Duksan Chemical Co., (Ansan, Republic of Korea). DEGME was purchased from Gattefosse (Saint-Priest, France), and double-distilled water was generated in the laboratory using a Millipore Milli-Q purification system (Millipore Corp., Milan, Italy). Polyvinylpyrrolidone K30 (PVP K30, *M_w_* = 44,000–54,000) provided by BASF SE (Ludwigshafen, Germany) was used as a stabilizer for the preparation of MHY498 nanosuspensions. Detailed information on MHY498 and its solvents is provided in Table S1.

### 2.2. Measurement of MHY498 Solubility

The solubility of MHY498 was determined in various monosolvents and (DEGME + water) solvent mixtures over the temperature range 293.15–313.15 K using the solid–liquid equilibrium technique [19,20]. Solvent mixtures were prepared with DEGME mass fractions ranging from 0.0 to 1.0 in 0.1 increments. An excess of MHY498 was added to each vial containing a monosolvent or (DEGME + water) solvent mixture, stirred, and placed in a water bath (BS-21, Jeiotech Co., Ltd., Daejeon, Republic of Korea) for 24 h to reach saturation, following conditions established in previous studies. The saturated solutions were filtered through a syringe filter, and the filtrate was diluted with ethanol to a concentration suitable for quantitative analysis. MHY498 concentrations were determined via UV spectrophotometry (8453 UV-vis, Agilent, Santa Clara, CA, USA) at 376 nm using quartz cuvettes with a 2 mm path length. A calibration curve was prepared with standard solution ranging from 1 to 10 μg∙mL^−1^ and exhibited a correlation coefficient of <0.999. All solubility measurements were performed in triplicate (*n* = 3), and the reported values are expressed as mean ± standard deviation. The mole fraction solubility (*x*_e_) of MHY498 in the monosolvent was calculated using Equation (1).

(1) $$x_{e}=\frac{m_{1}/M_{1}}{m_{1}/M_{1}+m_{2}/M_{2}}$$

where *m*_1_ and *m*_2_ are the masses of MHY498 in the solution and each monosolvent, respectively. *M*_1_ and *M*_2_ are the molecular weight of MHY498 (237.23 g∙mol^−1^) and each monosolvent, respectively.

The mole fraction solubility of MHY498 in the solvent mixture containing DEGME and water was calculated using Equation (2).

(2) $$x_{e}=\frac{m_{1}/M_{1}}{m_{1}/M_{1}+m_{2}/M_{2}+m_{3}/M_{3}}$$

where *m*_1_ and *M*_1_ are the same as in Equation (2); *m*_2_ and *m*_3_ represent the masses of DEGME and water in the mixture, respectively. *M*_2_ and *M*_3_ refers to the molecular weights of DEGME and water, respectively.

### 2.3. Powder X-Ray Diffractometry (PXRD)

MHY498 powder recovered from the saturated solution was analyzed using X-ray diffractometry (XRD) to determine whether any polymorphic transitions occurred during the solubility measurement. The XRD diffraction patterns were obtained at diffraction angles ranging from 5 to 50° using XPert X-ray diffractometer (40 kV voltage and 40 mA current, Malvern Panalytical, Almelo, The Netherlands) with Cu-Kα radiation and setting the scanning speed and step sizes of 3°∙min^−1^ and 0.01°, respectively.

### 2.4. Preparation of MHY498 Nanosuspension

MHY498 nanosuspensions were prepared based on solubility data and the Box–Behnken design to evaluate formulation feasibility and optimize nanosuspension properties [21,22,23,24,25]. The antisolvent precipitation method was applied using DEGME as the solvent and water as the antisolvent [19,20,26]. PVP K30 was incorporated into the aqueous antisolvent phase as a polymeric stabilizer during nanosuspension preparation. The polymer adsorbs onto the surface of precipitating drug particles, forming a stabilizing layer that limits particle–particle interactions during nucleation and early growth stages. This stabilization contributes to controlled particle formation and helps maintain colloidal stability of the nanosuspension. The selection and concentration range of PVP were determined based on preliminary studies and relevant literature. MHY498 was fully dissolved in DEGME and added to water containing PVP K30, with continuous stirring at 500 rpm until a homogeneous dispersion was achieved [18,22,23,24]. Particle size of the nanosuspension was measured by dynamic light scattering using a particle size analyzer (ELSZ-1000, Otsuka Electronics, Osaka, Japan). The Box–Behnken design was applied to efficiently evaluate the effects of the drug concentration (mole fraction) (X_1_), PVP K30 concentration (X_2_), and solvent-to-antisolvent ratio (X_3_) on the particle size of the nanosuspension (Y) during nanosuspension preparation. The variable levels (X_1_, X_2_, and X_3_) were set based on preliminary studies and solubility data. The Box–Behnken design and its statistical analysis were performed using Design Expert^®^ 11.0 (Stat-Ease, Inc., Minneapolis, MN, USA).

### 2.5. In Vivo SKIN Permeation Study

The skin permeability of MHY498 nanosuspensions was compared with that of MHY microsuspension using a transdermal diffusion system equipped with a vertical static Franz diffusion cell (FDC-6T, Logan Instrument Co., Somerset, NJ, USA). Nanosuspensions with particle sizes of 28.1 nm and 142.9 nm were prepared using the antisolvent precipitation method described in Section 2.4. Microsuspension was prepared by uniformly dispersing MHY498 in an aqueous solution containing DEGME and PVP K30, identical to the nanosuspension composition, resulting in an average particle size of approximately 2.1 μm.

The in vivo permeation study was approved by the Kyungsung University Ethics Committee (Approval No. 2016-010) and conducted in accordance with the institutional guidelines for the care and use of laboratory animals. All procedures were performed in compliance with internationally accepted ethical standards, including the ARRIVE guidelines and relevant IACUC principles. Sprague-Dawley rats were anesthetized, and their abdominal skin was shaved and surgically excised. Subcutaneous fat was carefully removed, and the skin samples (diffusion area of 0.635 cm^2^) were soaked in a phosphate buffer solution (pH 7.4) for approximately 3 h before the experiment. The skin membranes were then mounted between the donor and receptor chambers tightly fixed using a pinch clamp. The receptor chamber was filled with phosphate buffer and stirred magnetically at 600 rpm to ensure uniform distribution of permeated drug, while maintaining the temperature at 32 ± 2 °C. Samples containing 300 µg of MHY498 were applied to the donor chamber. At 0.25, 0.5, 1, 2, 3, 4, 6, 8, 12, and 24 h, 3 mL of receptor fluid was withdrawn and replaced with an equal volume of fresh phosphate buffer. Collected samples were filtered through a 0.45 µm syringe filter, and the amount of permeated drug was quantified using HPLC system (Shimadzu Corporation, Kyoto, Japan). HPLC analysis was conducted on a Kinetex 5u EVO C18 column (4.6 mm I.D × 250 mm, 5 μm; Phenomenex, Torrance, CA, USA) maintained at 40 °C, using a mobile phase of ethanol and distilled water (30:70, *v*/*v*) at a flow rate of 1.0 mL/min with an injection volume of 10 μL, and detection was performed with a UV–Vis detector set at 376 nm. Each experiment was performed in triplicate with 12 samples (*n* = 12) [27,28,29].

### 2.6. Data Analysis

Data were presented as mean ± standard deviation (SD). One-way analysis of variance (ANOVA) was performed to determine statistical significance between groups, and the Student-Newman-Keuls (SNK) test was performed for post hoc comparisons. The SPSS statistics software (IBM SPSS Statistics version 29.0, IBM Consulting, Armonk, NY, USA) was used for data analysis, and *p* values < 0.05 was considered statistically significant [27,30].

## 3. Results and Discussion

### 3.1. PXRD Data of MHY498

**Figure 2:**
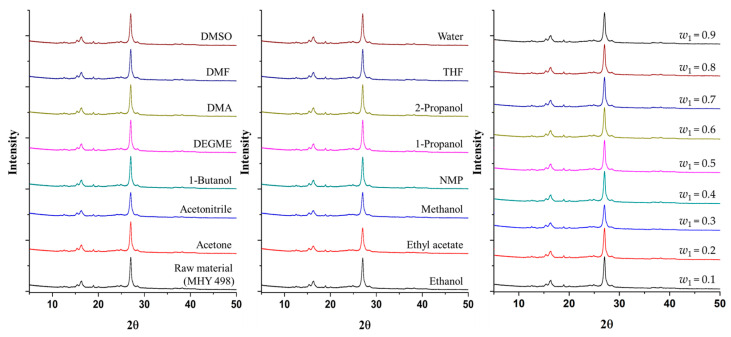
PXRD patterns of raw material and equilibrated MHY498 in 15 monosolvents and DEGME (1) + water (2) mixtures at 298.15 K.

The MHY498 recovered from various monosolvents and (DEGME + water) solvent mixtures after equilibration exhibited characteristic peaks at 2θ = 15.37, 16.35, and 27.05° (Figure 2), consistent with previously reported values [4]. Because these diffraction patterns were identical to those of raw MHY498, no crystalline changes occurred during the solubility measurement. The diffraction patterns of the recovered samples exhibited no additional peaks, peak shifts, or peak disappearance when compared with those of the raw MHY498. These observations indicate that the solubility measurement did not induce any polymorphic transformation and that MHY498 maintained its original crystalline form under the experimental conditions.

### 3.2. Solubility Data

#### 3.2.1. MHY498 Solubility in Various Monosolvents

**Figure 3:**
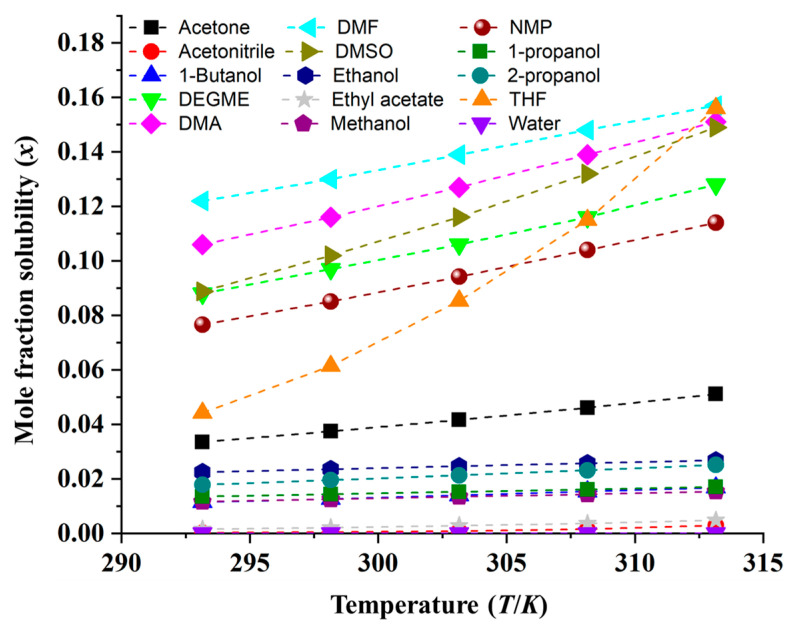
Mole fraction solubility of MHY498 (*x*) in 15 monosolvents at 293.15–313.15 K.

The mole fraction solubilities of MHY498 in various monosolvents, including acetone, acetonitrile, 1-butanol, DEGME, DMA, DMF, DMSO, ethanol, ethyl acetate, methanol, NMP, 1-propanol, 2-propanol, THF, and water, were determined over the temperature range 293.15–313.15 K (Table 1, Figure 3). In all solvents, the solubility of MHY498 increased with temperature. In particular, solubility in THF decreased sharply with decreasing temperature. In particular, the solubility measured in THF at 313.15 K increased almost similarly to that of DMF, which exhibited the highest solubility. At 298.15 K, the highest solubility was observed in DMF (1.30 × 10^−1^, at 298.15 K), which was approximately 20,968 times higher than that of water (6.20 × 10^−6^), which had the lowest solubility. The MHY498 solubility for monosolvent at 298.15 K was ranked in the following order: DMF > DMA > DMSO > DEGME > NMP > THF > acetone > ethanol > 2-propanol > 1-propanol > 1-butanol > methanol > ethyl acetate > acetonitrile > water.

The solubility of MHY498 was generally higher in polar aprotic solvents, such as DMF, DMA, DMSO, and NMP, than in the five alcoholic solvents, likely due to strong solute–solvent interactions involving the hydrogen bond acceptor groups of the solvents and the two hydroxyl groups (-OH) and one amide group (-NH) of MHY498. In contrast, the solubility of MHY498 in the five alcoholic solvents showed no consistent correlation with polarity or dielectric constant (methanol > ethanol > 1-propanol > 2-propanol > 1-butanol). These solubility values did not have a clear relationship with the physical properties of the solvent, such as polarity index (*E*_T_), dipole moments (*μ*), dielectric constants (*ε*), and Hildebrand solubility parameters (*δ*_H_). Overall, the solubility behavior of MHY498 was not only affected by temperature but was also linked to different factors, such as various solute–solvent or solvent–solvent interactions.

#### 3.2.2. MHY498 Solubility in (DEGME + Water) Mixed Solvents

In this study, DEGME was selected from monosolvents exhibiting high solubility for MHY498, and the solubility behavior of MHY498 was further investigated in (DEGME + water) solvent mixtures. Based on preliminary studies, when DMF, DMA, and DMSO were used as solvents in antisolvent precipitation method, rapid and uniform supersaturation was not effectively achieved upon mixing with the aqueous phase. This behavior was attributed to strong solvent–solute interactions and relatively slow solvent diffusion, which delayed the development of supersaturation. As a result, nucleation was limited and particle growth was favored, leading predominantly to the formation of micrometer-sized particles (Table S2). By contrast, DEGME exhibited solubility comparable to that of other polar solvents, indicating sufficient solubilization capacity for MHY498. Beyond its solubilizing ability, it demonstrated favorable performance in antisolvent precipitation method-based nanosuspension preparation. DEGME enabled rapid solvent diffusion into the aqueous phase, promoting instantaneous supersaturation and efficient nucleation. Moreover, DEGME is widely used in topical formulations because it can be used alone or in combination with other additives to reduce the barrier function of the stratum corneum without damaging the skin structure, thereby facilitating skin penetration [31,32,33]. Consequently, the DEGME–water system facilitated efficient nucleation and reproducible formation of nanosized particles, and was therefore selected as a formulation-relevant solvent for further nanosuspension development.

**Figure 4:**
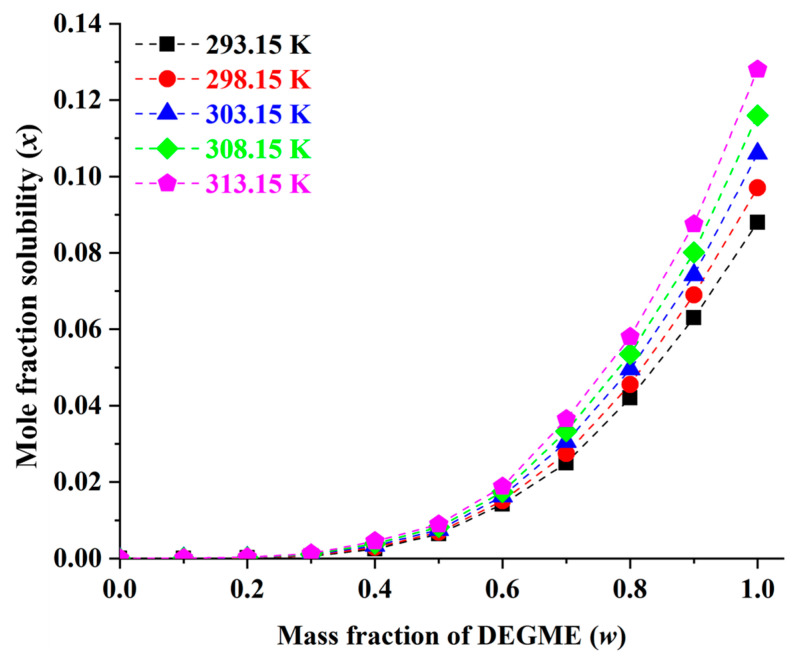
Mole fraction solubility of MHY498 (*x*) in DEGME (1) + water (2) mixture at 293.15–313.15 K.

The solubility data (*x*_e_) for MHY498 in (DEGME + water) mixtures were determined at five temperatures (293.15, 298.15, 303.15, 308.15, and 313.15 K) and are presented in Table 2 and Figure 4. At 303.15 K, the mole fraction solubility of MHY498 in pure water and DEGME at 303.15 K was determined as 6.95 (±0.11) × 10^−6^ and 1.06 (±0.02) × 10^−1^, respectively, indicating that the MHY498 solubility in DEGME was approximately 15,252-fold higher than that in pure water. Across all mixtures, the solubility of MHY498 increased with both temperature and the mole fraction of DEGME. As with monosolvents, no literature comparison was made because solubility data for MHY498 in {DEGME (1) + water (2)} mixture have not been previously reported. The observed solubility data in DEGME–water mixtures is relevant to formulation development for topical pharmaceutical or cosmetic applications. The solubility data obtained in these solvent mixture systems provide practical guidance for defining feasible compositions compatible with both nanosuspension preparation and topical application requirements. Accordingly, these results should be regarded as formulation-enabling information that supports solvent selection and formulation feasibility, rather than as a direct measure of intrinsic biological activity.

### 3.3. Effect of Solvent on Solubility

Solubility measurements were first performed to identify solvents exhibiting high solubility for MHY498, and the resulting experimental data were subsequently used to analyze solvent–solute interactions using the KAT-LSER model. This mechanistic insight provides a basis for subsequent thermodynamic correlation of the solubility data. The KAT-LSER model was used to describe the types of solute–solvent interactions and their energy contributions, which include solvatochromatic parameters, such as hydrogen bond donor acidity (*α*), hydrogen bond acceptor basicity (*β*), dipolarity/polarizability (π*), and Hildebrand solubility parameter (*δ*_H_) [34,35,36,37,38,39,40]. The model can be expressed as follows:

(3) $$ln\left(x_{e}\right)=c_{0}+c_{1}\alpha +c_{2}\beta +c_{3}\pi^{*}+c_{4}\left(\frac{V_{s}\delta_{H}^{2}}{100RT}\right)$$

where *V*_s_ indicates the molar volume of MHY498 estimated using Fedors’ method (133.7 cm^3^·mol^−1^) (Table S3). *R* refers to the ideal gas constant (8.314 J∙K^−1^∙mol^−1^) and *T* indicates the absolute temperature (298.15 K). *c*_0_ is a constant for the properties of the solute. c_1_ and c_2_ refer to the sensitivity of the solute to the solute–solvent interactions by hydrogen bonding. *c*_3_ and *c*_4_ pertain to the sensitivity of the solute to the nonspecific electrostatic solute–solvent and the solvent–solvent. The model was analyzed by performing multiple linear regression analysis (IBM SPSS Statistics 26, IBM Corporation, Armonk, NY, USA) using the solubility data of MHY498 in monosolvents at 298.15 K. The solvatochromatic parameters (*α*, *β*, and π*) for monosolvent are summarized in Table S4, and DEGME was excluded as no data related to the parameter were reported. Based on solution theory, *c*_1_ and *c*_2_ must be positive because of the favorable hydrogen bond interactions that promote dissolution. The most suitable regression equations with standard errors are presented as follows, according to statistical theory, such as *F* value, squared correlation coefficients (*R*^2^), and residual square sum (*RSS*) (Table S5) [41,42]:

(4) $$ln\left(x_{e}\right)=-8.66\left(2.13\right)+6.76\left(2.21\right)\beta +4.48\left(2.06\right)\pi^{*}-9.54(1.74)\left(\frac{V_{s}\delta_{H}^{2}}{100RT}\right)$$

whereas *n* = 14, *R*^2^ = 0.82, *RSS* = 17.44, and *F* = 15.24.

Therefore, hydrogen bond interactions of the solvent as an acceptor with MHY498 as the donor and nonspecific dipolarity/polarizability interactions could increase the solubility of MHY498. However, the solubility of MHY498 decreased with increasing self-cohesiveness (or structuredness) and solvent–solvent interactions. The relative contributions of *β*, π*, and *δ*_H_ to the solubility of MHY498 were 32.53%, 21.56%, and 45.91%, respectively, based on the estimated values. The hydrogen bond acceptor basicity (*β*), and Hildebrand solubility parameter (*δ*_H_) had a significant effect on the solubility of MHY498.

### 3.4. Correlation of MHY498 Solubility

To quantitatively describe the temperature dependence of MHY498 solubility, the experimentally determined solubility data were correlated using various thermodynamic and semi-empirical models. These approaches enable quantitative description and predictive evaluation of solubility behavior under varying thermal conditions and providing formulation- and process-relevant insights beyond simple experimental comparison.

#### 3.4.1. Evaluation of MHY498 Solubility in 15 Monosolvents

Various models, such as the van’t Hoff and modified Apelblat models, were used to evaluate the mole fraction solubility of MHY498 measured in the tested monosolvents [20,43]. The model parameters obtained by the fitting are listed in Table 3. The van’t Hoff model was applied to determine the linear relationship between the solubility of a solute (MHY498) in a solvent and the investigated temperature (*T*) and is expressed as follows:

(5) $$lnx_{e}=A+\frac{B}{T}$$

where *x*_e_ is the molar solubility of MHY498. Meanwhile, *A* and *B* are empirical parameters calculated by performing regression analysis on the obtained data.

The modified Apelblat model was used to explain the dependence of solubility (*x*_e_) on temperature, as shown in (6).

(6) $$lnx_{e}=A+\frac{B}{T}+C\ln T$$

where *A*, *B*, and *C* represent the constant parameters of this model.

#### 3.4.2. Evaluation of MHY498 Solubility in Binary Solvents

MHY498 solubility data in binary mixed solvents were fitted by applying van’t Hoff, modified Apelblat, CNIBS/R-K, Jouyban–Acree, Sun, Ma, modified Wilson, MRS, and Yalkowsky-Roseman models, and the values estimated through model fitting analysis are shown in Table 4, Table 5, Table 6, Table 7, Table 8 and Table 9 [44,45,46,47,48,49]. The van’t Hoff and modified Apelblat models are presented in Section 3.4.1.

The combined nearly ideal binary solvent/Redich–Kister (CNIBS/R-K) model was used to determine the relationship between the composition of the binary mixed solvent and solubility of the solute (MHY498) measured at the same temperature, as shown in Equation (7).

(7) $$lnx_{e}=B_{0}+B_{1}x_{A}+B_{2}x_{A}^{2}+B_{3}x_{A}^{3}+B_{4}x_{A}^{4}$$

where the model parameters *B*_0_, *B*_1_, *B*_2_, *B*_3_, and *B*_4_ were obtained by performing nonlinear regression analysis and *x*_A_ is the initial mole fraction of DEGME in the (DEGME + water) mixed solvent without MHY498.

Additionally, the Jouyban–Acree, Jouyban–Acree-van’t Hoff, and Jouyban–Acree-modified Apelblat, Ma, and Sun models were applied to confirm the correlation between the solubility of MHY498 and both the measured temperature and initial composition of the solvent mixture. The Jouyban–Acree model can predict the solubility of the solute (MHY498) in mixed solvents by describing the thermodynamic correlation of temperature and solvent composition with the measured solubility data and is expressed as Equation (8).

(8) $$lnx_{e}=w_{1}lnx_{1}+w_{2}lnx_{2}+\frac{w_{1}w_{2}}{T}\sum_{i=0}^{2}J_{i}{\left(w_{1}-w_{2}\right)}^{i}$$

where *J*_i_ terms containing *J*_1_, *J*_2_, and *J*_3_ are model parameters calculated by regression analysis of ($lnx_{e}-w_{1}lnx_{1}-w_{2}lnx_{2}$) against $\frac{w_{1}w_{2}}{T}$, $\frac{w_{1}w_{2}(w_{1}{-w}_{2})}{T}$, and $\frac{w_{1}w_{2}{\left(w_{1}{-w}_{2}\right)}^{2}}{T}$, respectively, and represent the molecular interactions between the solute and the solvent. *x*_e_ is the mole fraction solubility of MHY498 in a (DEGME + water) mixed solvent at a set temperature, *x*_1_ and *x*_2_ represent the solubilities of MHY498 measured in a solution containing DEGME and water alone, respectively, in a mixed solvent. *w*_1_ and *w*_2_ are the mass fractions of DEGME and water, respectively, in the mixed solvent.

The Jouyban–Acree model combined with the van’t Hoff model or modified Apelblat model can be applied to predict the solubility of solutes (MHY498) in binary solvent mixtures of various compositions more accurately. The Jouyban–Acree-van’t Hoff model can be expressed as follows:

(9) $$lnx_{e}=w_{1}\left(A_{1}+\frac{B_{1}}{T}\right)+w_{2}\left(A_{2}+\frac{B_{2}}{T}\right)+\frac{w_{1}w_{2}}{T}\sum_{i=0}^{2}J_{i}{\left(w_{1}-w_{2}\right)}^{i}$$

where the model parameters *A*_1_, *B*_1_, *A*_2_, *B*_2_, and *J_i_* were obtained by applying regression analysis. A simplified version of this model, the Sun model, was derived, and its general expression is shown in Equation (10).

(10) $$lnx_{e}=D_{1}+\frac{D_{2}}{T}+D_{3}w_{1}+D_{4}\frac{w_{1}}{T}+D_{5}\frac{w_{1}^{2}}{T}+D_{6}\frac{w_{1}^{3}}{T}+D_{7}\frac{w_{1}^{4}}{T}$$

where *D*_1_–*D*_7_ are the parameters in the simplified Sun model.

The Jouyban–Acree-modified Apelblat model combined for the same reason can be expressed as follows:

(11) $$lnx_{e}=w_{1}\left[A_{1}+\frac{B_{1}}{T}+C_{1}lnT\right]+w_{2}\left[A_{2}+\frac{B_{2}}{T}+C_{2}lnT\right]+\frac{w_{1}w_{2}}{T}\sum_{i=0}^{2}J_{i}{\left(w_{1}-w_{2}\right)}^{i}$$

where *A*_1_, *B*_1_, *C*_1_, *A*_2_, *B*_2_, *C*_2_, and *J_i_* are computed model parameters. The simplified Ma model is proposed in Equation (12).

(12) $$lnx_{e}=E_{1}+\frac{E_{2}}{T}+E_{3\;}lnT+E_{4}w_{1}+E_{5}\frac{w_{1}}{T}+E_{6}\frac{w_{1}^{2}}{T}+E_{7}\frac{w_{1}^{3}}{T}+E_{8}\frac{w_{1}^{4}}{T}+E_{9}w_{1}lnT$$

where *E*_1_ to *E*_9_ are the constants of Ma model.

The modified Wilson model is a nonlinear model describing solubility in non-aqueous and aqueous mixed solvents at a specific temperature, which can be expressed as Equation (13).

(13) $$-\ln x_{e}=1-\frac{w_{1}(1+\ln x_{1})}{w_{1}+w_{2}\lambda_{12}}-\frac{w_{2}(1+\ln x_{2})}{w_{1}\lambda_{21}+w_{2}}$$

where *λ*_12_ and *λ*_21_ represent equation constants parameters estimated by performing a regression analysis using solubility data.

The MRS model, which is used to correlate solubility at isothermal conditions through simple regression analysis, is described by Equation (14).

(14) $$lnx_{e}=\beta_{1}{w^{\prime }}_{1}+\beta_{2}{w^{\prime }}_{2}+\beta_{3}\left(\frac{1}{{w^{\prime }}_{1}}\right)+\beta_{4}\left(\frac{1}{{w^{\prime }}_{2}}\right)+\beta_{5}{w^{\prime }}_{1}{w^{\prime }}_{2}$$

where *β*_1_–*β*_5_ express model’s parameters. *w*^′^_1_ and *w*^′^_2_ can be calculated as *w*^′^_1_ = 0.96*w*_1_ + 0.02 and *w*^′^_2_ = 0.96*w*_2_ + 0.02.

The Yalkowsky–Roseman model was applied to predict the natural logarithmic solubility of MHY498 in (DEGME + water) mixed solvents using the solubility values of pure solvents 1 (DEGME) and 2 (water), as shown in Equation (15). The terms in this model employ the same definitions as in the previous equation.

(15) $$lnx_{e}=w_{1}lnx_{1}+w_{2}lnx_{2}$$

#### 3.4.3. Evaluation of MHY498 Solubility Data

The fitting accuracy of the solubility models was compared by calculating the relative average deviation (RAD) and root mean square deviation (RMSD), as defined in Equations (16) and (17), respectively, with *N* representing the number of data points.

(16) $$RAD=\frac{1}{N}\sum_{i=1}^{N}\left(\frac{\left|x_{c}-x_{e}\right|}{x_{e}}\right)$$

(17) $$RMSD={\left[\frac{1}{N}{\sum_{i=1}^{N}\left(x_{c}-x_{e}\right)}^{2}\right]}^{1/2}$$

where *x*_e_ and *x*_c_ are the experimental and calculated solubilities, respectively, of MHY498.

In this study, the correlation of solubility data with the temperature of the solvent was confirmed using various models. The fitting results derived by applying the van’t Hoff and modified Apelblat models to the solubility data measured in the monosolvents are presented in Table 3. The overall RAD values for van’t Hoff and modified Apelblat models were 2.47 × 10^−3^ and 2.24 × 10^−3^, respectively. The overall RMSD values for van’t Hoff and modified Apelblat models were 1.07 × 10^−4^ and 8.85 × 10^−5^, respectively. These results indicate strong agreement and correlation between the calculated and experimental data, demonstrating that both models provided satisfactory solubility correlation. Additionally, nine different models were applied to fit the solubility data of MHY498 in (DEGME + water) mixed solvent, and the results are presented in Table 4, Table 5, Table 6, Table 7, Table 8 and Table 9. The overall RAD values for each model were as follows: van’t Hoff (3.97 × 10^−3^), modified Apelblat (2.64 × 10^−3^), CNIBS/R-K (3.12 × 10^−1^), Jouyban–Acree (3.97 × 10^−2^), Sun (4.05 × 10^−2^), Ma (4.05 × 10^−2^), modified Wilson (4.24 × 10^−2^), MRS (4.01 × 10^−2^), and Yalkowsky–Roseman (6.31 × 10^−1^). The overall RMSD values for each model were as follows: van’t Hoff (1.04 × 10^−4^), modified Apelblat (2.66 × 10^−2^), CNIBS/R-K (7.00 × 10^−3^), Jouyban–Acree (7.36 × 10^−4^), Sun (7.90 × 10^−4^), Ma (7.69 × 10^−4^), modified Wilson (1.05 × 10^−3^), MRS (1.60 × 10^−3^), and Yalkowsky–Roseman (1.71 × 10^−2^). These results demonstrate that the solubility data also exhibited good correlations for the nine additionally applied models, indicating their applicability for predicting the solubility of MHY498 in these solvents.

### 3.5. Thermodynamic Properties for MHY498 Dissolution in the Studied Solvents

The thermodynamic properties of the experimental solubility data were analyzed to understand the dissolution behavior of MHY498 in various solvents in a solid–liquid equilibrium system. The properties, including the dissolution enthalpy (∆*H*°), Gibbs free energy (∆*G*°), and dissolution entropy (∆*S*°) for dissolution of MHY498, can be explained based on the van’t Hoff equation, which is an empirical model [50,51,52,53,54]. This is expressed by Equation (18) and is calculated from the slope and intercept estimated by plotting the ln *x* values according to (1/*T* − 1/*T_hm_*).

(18) $$\left(\frac{\partial \ln x}{\partial (1/T-1/T_{hm})}\right)=-\frac{∆H^{{}^{\circ}}}{R}$$

where *R* indicates the gas constant (8.314 J∙mol^−1^∙K^−1^). *T*_hm_ indicates the harmonic mean temperature (302.99 K), defined by Equation (19).

(19) $$T_{hm}=n/\sum_{n=1}^{n}\left(1/T\right)$$

The ∆*G*° for dissolution of MHY498 at 302.99 K was calculated using Equation (20), and ∆*S*° was subsequently estimated from ∆*H*° and ∆*G*° using Equation (21) to evaluate the standard change for entropy during the dissolution process.

(20) $$\Delta G^{{}^{\circ}}=-RT_{hm}\times intercept$$

(21) $$∆S^{{}^{\circ}}=\frac{∆H^{{}^{\circ}}-\Delta G^{{}^{\circ}}}{T_{hm}}$$

The enthalpy (%*ξ_H_*) and entropy (*%ξ_TS_*) were identified using Equations (20) and (21) to confirm the relative contribution of ∆*H*° and ∆*S*° to the change in Gibbs energy during the dissolution of MHY498, and the formulas are as follows.

(22) $${\%\xi }_{H}=\frac{{|∆H}^{{}^{\circ}}|}{\left|{∆H}^{{}^{\circ}}\right|+\left|T\Delta S^{{}^{\circ}}\right|}\times 100$$

(23) $${\%\xi }_{TS}=\frac{\left|T\Delta S^{{}^{\circ}}\right|}{\left|{∆H}^{{}^{\circ}}|+|T\Delta S^{{}^{\circ}}\right|}\times 100$$

The results of the thermodynamic properties (∆*H*°, ∆*G*°, and ∆*S*°) and the relative contributions (%*ξ_H_* and *%ξ_TS_*) analyzed for the dissolution behavior of MHY498 in a monosolvent at 302.99 K are shown in Table 10. The thermodynamic properties obtained for the 15 selected monosolvents were all positive, indicating that the dissolution of MHY498 in these solvents was endothermic and driven by entropy. This observation aligns with the fact that MHY498 solubility decreases as the temperature decreases. Comparing %*ξ_H_* and *%ξ_TS_* for the corresponding monosolvents, the minimum values of %*ξ_H_* and *%ξ_TS_* were 53.47% and 0.40%, respectively, and the value of %*ξ_H_* was greater than that of *%ξ_TS_*. ∆*H*° was found to have a more significant effect on ∆*G*° than ∆*S*°.

Table 11 presents the thermodynamic properties analyzed for the dissolution of MHY498 in the DEGME + water mixtures at 302.99 K. The ∆*H*° and ∆*G*° values were positive and ranged from 10.72 to 30.08 kJ∙mol^−1^ and 5.65 to 29.95 kJ∙mol^−1^, respectively. The ∆*S*° values ranged from 0.40 to 28.57 J∙mol^−1^∙K^−1^. These results indicate that the dissolution of MHY498 in the (DEGME + water) solvent mixture was endothermic and entropy-driven and that the molecular interactions between the MHY498 and solvent molecules were considerably stronger than those among solvent molecules themselves. The %*ξ_H_* and *%ξ_TS_* values are also presented in Table 11, where *ξ_H_* ranged from 62.46% to 99.60% and *ξ_TS_* ranged from 0.40% to 37.54%. As in monosolvent, ∆*H*° was confirmed to have main effect on the change in Gibbs energy because the value of %*ξ_H_* was larger than that of *%ξ_TS_*.

### 3.6. Characterization of MHY498 Nanosuspension

**Figure 5:**
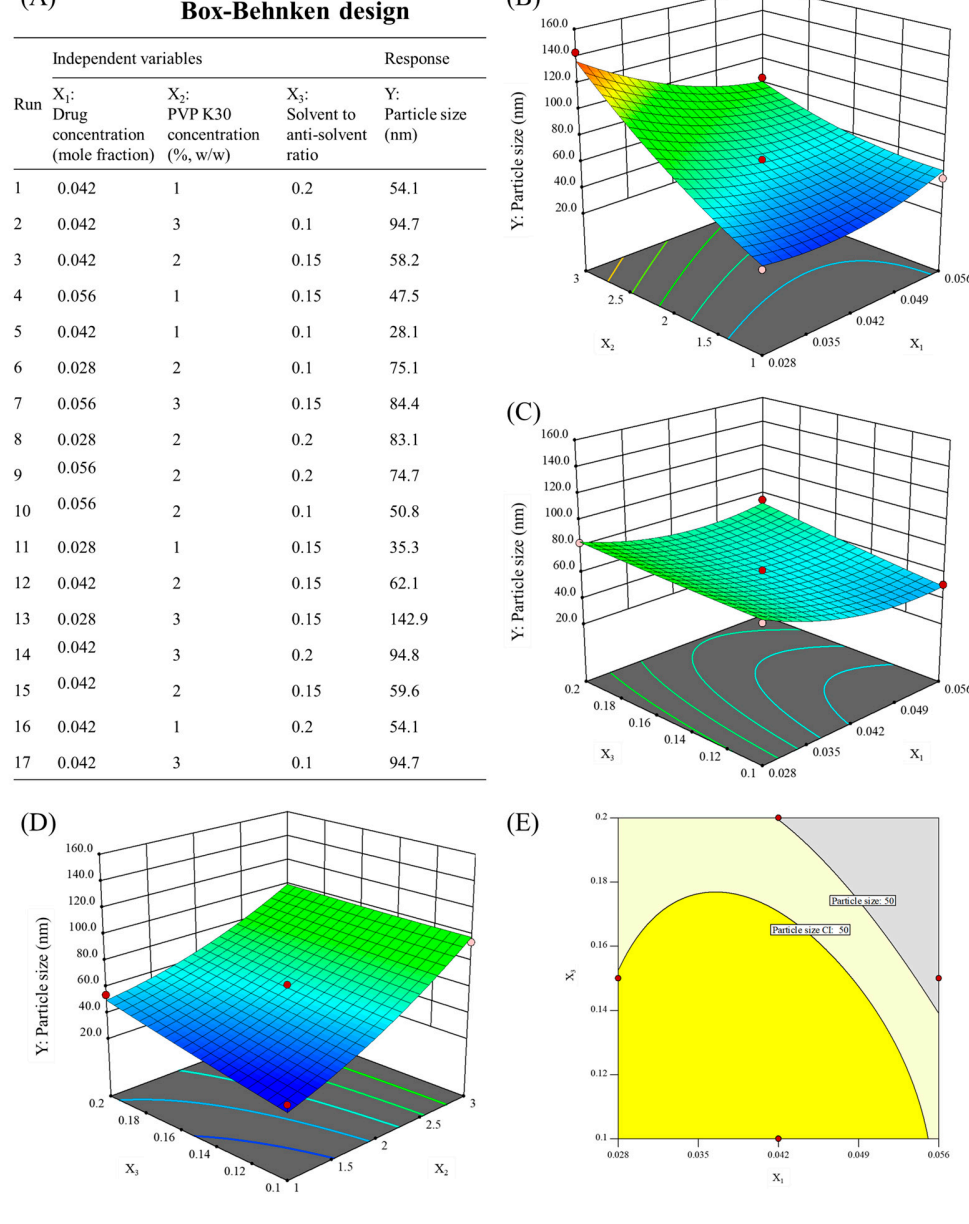
(**A**) Box–Behnken design matrix with experimental responses, (**B**–**D**) 3D response surface plots showing interaction between variables, and (**E**) optimized design space for particle sizes below 50 nm at X_2_ = 1. Particle size CI denotes the 50 nm criterion within the 95% confidence interval.

As shown in Figure 4, the solubility of MHY498 increased rapidly when the DEGME fraction in the mixture exceeded 0.2. For efficient nanosuspension preparation via solvent–antisolvent mixing, the solvent-to-antisolvent ratio was maintained below 0.2. To optimize the composition of MHY498 nanosuspensions, formulations were prepared under varying conditions based on a Box–Behnken design generated using Design Expert^®^ 11.0 (Stat-Ease, Inc., Minneapolis, MN, USA). The smallest particle size (28.1 nm) was achieved with a formulation containing a drug concentration of 0.042, PVP K30 concentration of 1, and a solvent-to-antisolvent ratio of 0.1, while all nanosuspensions prepared according to the design matrix showed particle sizes below 150 nm, as shown in Figure 5A. ANOVA confirmed that the statistical criteria were satisfied, as detailed in Table S6 [55,56,57,58,59]. The effects of drug concentration (X_1_), PVP K30 concentration (X_2_), and the antisolvent-to-solvent ratio (X_3_) on particle size are illustrated in the 3D response surface plot shown in Figure 5B–D.

The particle size of MHY498 nanosuspensions was significantly affected by the formulation and process variables, including drug concentration (X_1_), PVP K30 concentration (X_2_), and the antisolvent-to-solvent ratio (X_3_). The increase in particle size observed with increasing drug concentration (X_1_) can be attributed to supersaturation-driven growth behavior during the antisolvent precipitation method. At higher drug loadings, the rapid generation of high supersaturation favors accelerated particle growth following nucleation. Under these conditions, the available stabilizer is insufficient to effectively cover newly formed particle surfaces during the early stages of particle formation, shifting the system toward particle growth rather than the formation of uniformly sized nanoparticles [22,23]. Consequently, an overall increase in particle size is observed. The decrease in particle size with decreasing PVP K30 concentration (X_2_) suggests that, within the investigated range, the stabilizer concentration governs particle size by modulating the balance between nucleation and growth during the antisolvent precipitation method. Lower stabilizer levels reduced drug–stabilizer interactions in solution, thereby facilitating faster supersaturation development and promoting extensive nucleation. The resulting high nuclei density distributed the available solute among multiple growing entities, limiting subsequent crystal growth. In contrast, higher stabilizer concentrations suppressed nucleation and favored growth-dominated particle evolution, yielding larger particles [23,24,25,55]. These results indicate that a lower PVP K30 concentration is more favorable for achieving smaller particle sizes in the nanosuspension system and support the ANOVA results indicating X_2_ as the most statistically significant variable. A decrease in the antisolvent-to-solvent ratio (X_3_) also resulted in smaller particle sizes. At lower antisolvent-to-solvent ratios, rapid local solvent dilution upon mixing led to accelerated supersaturation development, favoring burst nucleation over subsequent crystal growth. The formation of a high density of nuclei distributed the available solute among multiple growing entities, thereby restricting particle growth. Accordingly, particle size can be tuned by controlling X_3_ to adjust the relative contributions of nucleation and post-nucleation growth during precipitation [22,24,25,26].

To further refine the design space, a stricter optimization criterion was set by requiring particle sizes below 50 nm. This criterion was selected as a performance-driven target because smaller particle sizes increase the effective surface area, can enhance dissolution behavior, and reduce aggregation risk in topical nanosuspension systems. Based on this criterion, the response surface model was used to delineate an optimized region within the design space, which was subsequently evaluated at a 95% confidence level (*α* = 0.05) to ensure statistical robustness. The statistically robust optimized region is highlighted in yellow in Figure 5E. For comparison, the light-yellow area in Figure 5E indicates the region satisfying the particle size criterion without applying the confidence level constraint. Although this unconstrained region meets the numerical requirement, it does not provide the same level of statistical confidence. Therefore, the final optimized region was selected as the largest continuous area meeting the 50 nm criterion within the 95% confidence interval. Under these optimized conditions, the PVP K30 concentration (X_2_) was fixed at 1%.

The optimized nanosuspension (particle size 28.1 nm) also exhibited good physical stability during storage at long-term condition (298.15 K, 60% RH) for up to 6 months, with no detectable particle growth or aggregation, supporting their practical applicability as topical formulations (Table S7).

### 3.7. Skin Permeation Study

**Figure 6:**
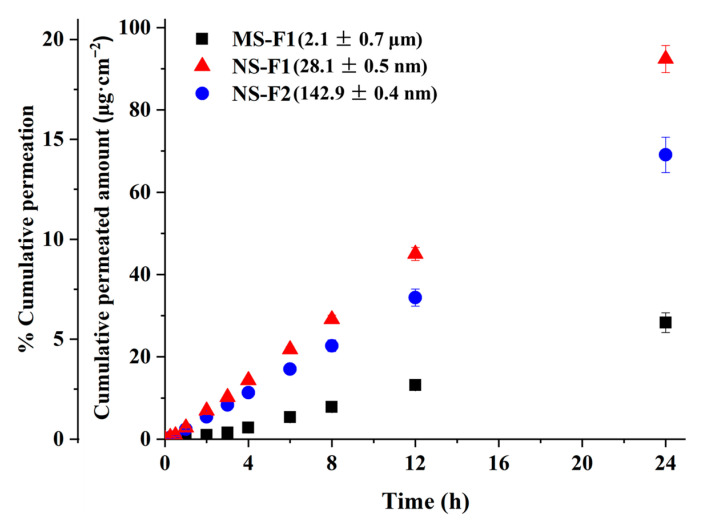
Cumulative permeation profile of MHY498 through the skin from a microsuspension (MS-F1) and nanosuspensions (NS-F1 and NS-F2) over 24 h (*n* = 12, mean ± SD).

A 24 h skin permeation study was conducted using nanosuspension and microsuspension formulations of MHY498 to evaluate their permeation characteristics. The raw material had an average particle size of approximately 5.9 ± 1.2 μm, and three different formulations were prepared by adjusting the particle size accordingly. From the compositions generated through the Box–Behnken design, two nanosuspensions were selected: NS-F1, which had the smallest particle size (28.1 ± 0.5 nm) and NS-F2, with the largest particle size (142.9 ± 0.4 nm). The microsuspension (MS-F1) was prepared from the same composition using a simple milling process, yielding a mean particle size of 2.1 ± 0.7 μm. All three formulations had an identical concentration of MHY498 and overall composition. The permeation profile of MHY498 from the nanosuspension and microsuspension formulations are presented in Figure 6. To analyze the permeation characteristics, the steady-state flux (*J*_ss_, μg·h^−1^·cm^−2^) was determined by calculating the slope of the linear portion of the graph using Equation (24):

(24) $$J=\frac{dm}{Sdt}$$

where *S* is the permeation area (cm^2^) [27,28,29,60].

In the permeation study using a Franz diffusion cell, the skin permeability of MHY498 was strongly influenced by the particle size of the formulation (*n* = 12). ANOVA showed that there were significant differences among the samples (*F*_2,33_=432.868, *p* < 0.05), which in order of increasing in both of the cumulative permeated amounts at 24 h and *J*_ss_ value of MHY498, were ranked by the SNK test (*p* < 0.05) as follows: microsuspension (MS-F1) < nanosuspension (NS-F2) < nanosuspension (NS-F1). In particular, the cumulative permeated amounts at 24 h for the nanosuspensions, NS-F1 and NS-F2 were 92.4 ± 3.3 μg·cm^−2^ and 69.1 ± 4.3 μg·cm^−2^, respectively. In contrast, the microsuspension, MS-F1, permeated approximately 28.3 ± 2.4 μg·cm^−2^ of the drug under the same conditions, which was about 2.4–3.3-fold lower than that of the nanosuspensions. The *J*_ss_ values further supported the enhanced permeation performance of the nanosuspensions. Both NS-F1 and NS-F2 showed higher flux rates, measured at 3.90 ± 0.21 μg·h^−1^·cm^−2^ and 2.90 ± 0.27 μg·h^−1^·cm^−2^, respectively, than only 1.28 ± 0.17 μg·h^−1^·cm^−2^ for MS-F1. When expressed as a percentage of the applied dose, the nanosuspension formulation (NS-F1) exhibited approximately 20% drug permeation over 24 h. This level of permeation is comparable to previously reported solid lipid nanoparticle-based formulations, for which cumulative permeation amount of approximately 100 µg·cm^−2^ over 24 h have been reported. The cumulative amount of drug permeated in the present study over the same time period was of a similar magnitude, despite differences in carrier composition [4]. This marked increase indicates that the nanosuspensions substantially improved drug permeation across the skin barrier. The improved permeation can be primarily attributed to the drastically reduced particle size in the nanosuspensions, which results in an increased surface area, leading to enhanced dissolution rates and increased concentration gradient driving diffusion. This enhanced permeation profile supports the potential application of nanosuspension formulations for topical drug delivery, where efficient and sustained skin absorption is critical for therapeutic efficacy [60,61,62,63,64].

## 4. Conclusions

In this study, the solubility of MHY498 in various monosolvents and in DEGME + water solvent mixtures was determined at 293.15, 298.15, 303.15, 308.15, and 313.15 K using the solid–liquid equilibrium method. Among the 15 monosolvents, the highest solubility of MHY498 was observed in DMF, DMA, DMSO, and DEGME, while the lowest solubility was observed in water. Analysis using the KAT-LSER model indicated that hydrogen bond interactions, with the solvent as the acceptor and MHY498 as the donor, as well as nonspecific dipolarity/polarizability interactions, contributed to increased solubility of MHY498. Conversely, higher solvent self-cohesiveness (or structuredness) and stronger solvent–solvent interactions led to decreased solubility. The solubility data of MHY498 were successfully correlated using the van’t Hoff, modified Apelblat, CNIBS/R-K, Jouyban–Acree, Sun, Ma, modified Wilson, MRS, and Yalkowsky–Roseman models, demonstrating that these models can reliably predict MHY498 solubility. Thermodynamic analysis indicated that dissolution in both monosolvents and DEGME + water mixtures was endothermic and spontaneous. Based on the solubility behavior in DEGME + water mixtures, MHY498 nanosuspensions with a particle size of 28.1 nm were successfully prepared using the antisolvent precipitation method under conditions optimized by the Box–Behnken design. These nanosuspension formulations exhibited significantly enhanced skin permeation compared with conventional microsuspensions and may serve as promising candidates for the development of topical dosage forms of MHY498.

## Figures and Tables

**Table 1:** Mole fraction solubility data (*x*_e_) of MHY498 in various monosolvents at 293.15–313.15 K.

Solvent	*x*_e_				
	*T* = 293.15 K	*T* = 298.15 K	*T* = 303.15 K	*T* = 308.15 K	*T* = 313.15 K
Acetone	3.35 (±0.05) × 10^−2^	3.74 (±0.04) × 10^−2^	4.16 (±0.04) × 10^−2^	4.61 (±0.02) × 10^−2^	5.11 (±0.04) × 10^−2^
Acetonitrile	2.01 (±0.06) × 10^−4^	4.13 (±0.06) × 10^−4^	7.95 (±0.04) × 10^−4^	1.51 (±0.06) × 10^−3^	2.91 (±0.03) × 10^−3^
1-Butanol	1.14 (±0.01) × 10^−2^	1.26 (±0.03) × 10^−2^	1.39 (±0.02) × 10^−2^	1.53 (±0.03) × 10^−2^	1.68 (±0.03) × 10^−2^
DEGME	8.80 (±0.01) × 10^−2^	9.70 (±0.03) × 10^−2^	1.06 (±0.02) × 10^−1^	1.16 (±0.03) × 10^−1^	1.28 (±0.02) × 10^−1^
DMA	1.06 (±0.02) × 10^−1^	1.16 (±0.03) × 10^−1^	1.27 (±0.05) × 10^−1^	1.39 (±0.01) × 10^−1^	1.51 (±0.03) × 10^−1^
DMF	1.22 (±0.02) × 10^−1^	1.30 (±0.05) × 10^−1^	1.39 (±0.04) × 10^−1^	1.48 (±0.04) × 10^−1^	1.57 (±0.02) × 10^−1^
DMSO	8.88 (±0.03) × 10^−2^	1.02 (±0.02) × 10^−1^	1.16 (±0.01) × 10^−1^	1.32 (±0.02) × 10^−1^	1.49 (±0.01) × 10^−1^
Ethanol	2.16 (±0.04) × 10^−2^	2.30 (±0.04) × 10^−2^	2.46 (±0.01) × 10^−2^	2.60 (±0.03) × 10^−2^	2.77 (±0.05) × 10^−2^
Ethyl acetate	1.53 (±0.05) × 10^−3^	2.06 (±0.03) × 10^−3^	2.77 (±0.05) × 10^−3^	3.61 (±0.03) × 10^−3^	4.77 (±0.04) × 10^−3^
Methanol	1.15 (±0.02) × 10^−2^	1.24 (±0.04) × 10^−2^	1.34 (±0.03) × 10^−2^	1.43 (±0.02) × 10^−2^	1.54 (±0.04) × 10^−2^
NMP	7.66 (±0.03) × 10^−2^	8.51 (±0.02) × 10^−2^	9.42 (±0.05) × 10^−2^	1.04 (±0.02) × 10^−1^	1.14 (±0.03) × 10^−1^
1-Propanol	1.32 (±0.02) × 10^−2^	1.41 (±0.03) × 10^−2^	1.52 (±0.01) × 10^−2^	1.63 (±0.05) × 10^−2^	1.74 (±0.03) × 10^−2^
2-Propanol	1.78 (±0.01) × 10^−2^	1.95 (±0.03) × 10^−2^	2.13 (±0.01) × 10^−2^	2.31 (±0.04) × 10^−2^	2.51 (±0.05) × 10^−2^
THF	4.42 (±0.06) × 10^−2^	6.14 (±0.04) × 10^−2^	8.54 (±0.05) × 10^−2^	1.15 (±0.02) × 10^−1^	1.56 (±0.04) × 10^−1^
Water	4.60 (±0.02) × 10^−6^	5.60 (±0.04) × 10^−6^	6.95 (±0.03) × 10^−6^	8.33 (±0.03) × 10^−6^	1.01 (±0.01) × 10^−5^

**Table 2:** Mole fraction solubility data (*x*_e_) of MHY498 in DEGME (1) + water (2) mixtures at 293.15–313.15 K.

*w*_1_	*x*_e_				
	*T* = 293.15 K	*T* = 298.15 K	*T* = 303.15 K	*T* = 308.15 K	*T* = 313.15 K
0.0	4.60 (±0.02) × 10^−6^	5.60 (±0.04) × 10^−6^	6.95 (±0.03) × 10^−6^	8.33 (±0.03) × 10^−6^	1.01 (±0.01) × 10^−5^
0.1	3.90 (±0.07) × 10^−5^	4.64 (±0.02) × 10^−5^	5.50 (±0.12) × 10^−5^	6.35 (±0.13) × 10^−5^	7.54 (±0.09) × 10^−5^
0.2	1.78 (±0.02) × 10^−4^	2.11 (±0.01) × 10^−4^	2.47 (±0.03) × 10^−4^	2.87 (±0.01) × 10^−4^	3.30 (±0.03) × 10^−4^
0.3	6.53 (±0.04) × 10^−4^	7.69 (±0.03) × 10^−4^	9.00 (±0.03) × 10^−4^	1.07 (±0.01) × 10^−3^	1.25 (±0.01) × 10^−3^
0.4	2.45 (±0.01) × 10^−3^	2.88 (±0.01) × 10^−3^	3.32 (±0.01) × 10^−3^	3.86 (±0.01) × 10^−3^	4.50 (±0.03) × 10^−3^
0.5	6.40 (±0.06) × 10^−3^	6.90 (±0.01) × 10^−3^	7.50 (±0.04) × 10^−3^	8.19 (±0.03) × 10^−3^	8.90 (±0.01) × 10^−3^
0.6	1.42 (±0.02) × 10^−2^	1.51 (±0.02) × 10^−2^	1.63 (±0.01) × 10^−2^	1.74 (±0.01) × 10^−2^	1.88 (±0.03) × 10^−2^
0.7	2.50 (±0.04) × 10^−2^	2.75 (±0.03) × 10^−2^	3.05 (±0.02) × 10^−2^	3.33 (±0.02) × 10^−2^	3.65 (±0.02) × 10^−2^
0.8	4.20 (±0.08) × 10^−2^	4.55 (±0.05) × 10^−2^	4.95 (±0.05) × 10^−2^	5.35 (±0.03) × 10^−2^	5.80 (±0.06) × 10^−2^
0.9	6.30 (±0.11) × 10^−2^	6.90 (±0.08) × 10^−2^	7.42 (±0.11) × 10^−2^	8.01 (±0.16) × 10^−2^	8.75 (±0.02) × 10^−2^
1.0	8.80 (±0.01) × 10^−2^	9.70 (±0.01) × 10^−2^	1.06 (±0.02) × 10^−1^	1.16 (±0.02) × 10^−1^	1.28 (±0.01) × 10^−1^

**Table 3:** Parameter values of van’t Hoff and modified Apelblat models for solubility data of MHY498 in the selected monosolvents.

Solvent	van’t Hoff Model				Modified Apelblat Model				
	*A*	*B*	RAD	RMSD	*A*	*B*	*C*	RAD	RMSD
Acetone	33.096	−12,195.7	1.01 × 10^−2^	1.60 × 10^−5^	−18.693	−946.707	3.261	5.13 × 10^−4^	2.54 × 10^−5^
Acetonitrile	3.202	−1934.6	7.80 × 10^−4^	4.41 × 10^−5^	−1.355	−10,641.348	5.131	9.80 × 10^−3^	1.44 × 10^−5^
1-Butanol	1.598	−1780.4	8.07 × 10^−4^	1.18 × 10^−5^	−23.327	−655.874	3.713	9.04 × 10^−5^	1.45 × 10^−6^
DEGME	3.380	−1703.8	3.49 × 10^−3^	4.46 × 10^−4^	−83.102	2198.045	12.881	2.12 × 10^−3^	2.59 × 10^−4^
DMA	3.320	−1631.5	1.25 × 10^−3^	1.69 × 10^−4^	−16.893	−719.574	3.011	1.08 × 10^−3^	1.60 × 10^−4^
DMF	1.867	−1164.4	9.77 × 10^−4^	1.51 × 10^−4^	−4.052	−897.354	0.882	1.02 × 10^−3^	1.51 × 10^−4^
DMSO	5.679	−2374.4	1.13 × 10^−3^	1.32 × 10^−4^	21.412	−3084.214	−2.343	8.76 × 10^−4^	1.18 × 10^−4^
Ethanol	0.048	−1138.549	1.86 × 10^−3^	5.14 × 10^−5^	−11.951	−497.159	1.787	1.66 × 10^−3^	5.01 × 10^−5^
Ethyl acetate	11.278	−5206.3	3.56 × 10^−3^	1.47 × 10^−5^	24.516	−5803.550	−1.972	3.57 × 10^−3^	1.51 × 10^−5^
Methanol	0.086	−1334.213	1.77 × 10^−3^	3.05 × 10^−5^	−5.464	−1083.842	0.827	1.72 × 10^−3^	3.02 × 10^−5^
NMP	3.670	−1828.7	8.02 × 10^−4^	1.05 × 10^−4^	26.093	−2840.390	−3.340	5.69 × 10^−4^	6.43 × 10^−5^
1-Propanol	0.038	−1280.515	1.77 × 10^−3^	3.38 × 10^−5^	−34.914	296.406	5.206	1.92 × 10^−3^	3.11 × 10^−5^
2-Propanol	1.339	−1573.4	8.79 × 10^−4^	2.28 × 10^−5^	14.927	−2186.428	−2.024	7.63 × 10^−4^	2.17 × 10^−5^
THF	16.612	−5784.2	3.12 × 10^−3^	3.78 × 10^−4^	42.129	−6935.405	−3.801	3.36 × 10^−3^	3.85 × 10^−4^
Water	0.048	−3617.526	4.74 × 10^−3^	3.63 × 10^−8^	−9.841	−3171.368	1.473	4.58 × 10^−3^	3.63 × 10^−8^

**Table 4:** Model parameters, *R*^2^, RAD, and RMSD values of the van’t Hoff and modified Apelblat models by fitting the solubility data.

*w*_1_	van’t Hoff Model					Modified Apelblat Model					
	*A*	*B*	*R* ^2^	RAD	RMSD	*A*	*B*	*C*	*R* ^2^	RAD	RMSD
0.0	0.048	−3617.526	1.000	4.74 × 10^−3^	3.63 × 10^−8^	−9.841	−3171.368	1.473	1.000	4.58 × 10^−3^	5.49 × 10^−3^
0.1	0.073	−2997.058	0.999	4.87 × 10^−3^	3.91 × 10^−7^	−23.238	−1945.341	3.472	0.999	5.07 × 10^−3^	6.02× 10^−3^
0.2	1.035	−2833.009	1.000	3.84 × 10^−3^	1.01 × 10^−6^	116.500	−8042.497	−17.198	1.000	3.70 × 10^−4^	3.15 × 10^−4^
0.3	2.860	−2990.132	0.999	4.80 × 10^−3^	5.03 × 10^−6^	−129.519	2982.518	19.718	1.000	2.76 × 10^−3^	2.74 × 10^−3^
0.4	3.436	−2796.911	0.999	4.41 × 10^−3^	1.77 × 10^−5^	−88.892	1395.718	13.752	1.000	3.26 × 10^−3^	2.04 × 10^−3^
0.5	0.142	−1524.570	0.998	4.56 × 10^−3^	3.70 × 10^−5^	−140.729	4831.210	20.983	1.000	1.60 × 10^−3^	6.08 × 10^−3^
0.6	0.139	−1289.807	0.997	4.78 × 10^−3^	8.40 × 10^−5^	−130.283	4594.500	19.426	0.999	2.25 × 10^−3^	1.43 × 10^−2^
0.7	2.250	−1741.324	1.000	2.09 × 10^−3^	7.81 × 10^−5^	9.650	−2075.198	−1.102	1.000	2.16 × 10^−3^	2.88 × 10^−2^
0.8	1.884	−1482.430	1.000	1.78 × 10^−3^	9.66 × 10^−5^	−45.939	675.240	7.123	1.000	9.29 × 10^−4^	4.91 × 10^−2^
0.9	2.284	−1479.878	0.998	4.33 × 10^−3^	3.74 × 10^−4^	−55.703	1136.382	8.637	0.999	3.89 × 10^−3^	7.14 × 10^−2^
1.0	3.380	−1703.826	0.999	3.49 × 10^−3^	4.46 × 10^−4^	−83.115	2198.618	12.883	1.000	2.12 × 10^−3^	1.06 × 10^−1^

**Table 5:** Model parameters, *R*^2^, RAD, and RMSD values of the simplified CNIBS/R-K model by fitting the solubility data.

*T*/K	Simplified CNIBS/R-K Model							
	*B*_0_	*B*_1_	*B*_2_	*B*_3_	*B*_4_	*R* ^2^	RAD	RMSD
293.15	−11.633	90.917	−348.465	534.329	−267.582	0.988	3.02 × 10^−1^	5.90 × 10^−3^
298.15	−11.430	89.679	−344.354	528.976	−265.209	0.988	3.10 × 10^−1^	6.39 × 10^−3^
303.15	−11.224	88.236	−338.472	519.573	−260.362	0.987	3.10 × 10^−1^	6.85 × 10^−3^
308.15	−11.045	87.410	−336.276	517.215	−259.462	0.987	3.17 × 10^−1^	7.51 × 10^−3^
313.15	−10.855	86.334	−332.659	512.370	−257.250	0.986	3.21 × 10^−1^	8.14 × 10^−3^

**Table 6:** Model parameters, *R*^2^, RAD, and RMSD values for MHY498 in (DEGME + water) mixed solvents obtained using solubility data.

Jouyban-Acree Model		Ma Model		Sun Model	
Parameter	Value	Parameter	Value	Parameter	Value
*J*_0_	2657.836	E_1_	−18.947	D_1_	0.524
*J*_1_	−813.099	E_2_	−2873.530	D_2_	−3752.014
*J*_2_	140.737	E_3_	2.900	D_3_	2.140
		E_4_	−67.767	D_4_	5787.286
		E_5_	8941.308	D_5_	−5497.349
		E_6_	−5497.363	D_6_	2398.038
		E_7_	2398.060	D_7_	−424.999
		E_8_	−425.010		
		E_9_	10.413		
*R* ^2^	0.995	R^2^	1.000	R^2^	1.000
RAD	3.97 × 10^−2^	RAD	4.05 × 10^−2^	RAD	4.05 × 10^−2^
RMSD	7.36 × 10^−4^	RMSD	7.69 × 10^−4^	RMSD	7.90 × 10^−4^

**Table 7:** Model parameters, *R*^2^, RAD, and RMSD values of the modified Wilson model by fitting the solubility data.

*T*/K	Modified Wilson Model				
	*λ*_12_	*λ*_21_	*R* ^2^	RAD	RMSD
293.15	2.219	2.076	1.000	1.13 × 10^−1^	1.53 × 10^−3^
298.15	2.037	2.110	1.000	9.47 × 10^−2^	1.22 × 10^−3^
303.15	1.990	2.122	1.000	8.61 × 10^−2^	1.54 × 10^−3^
308.15	1.822	2.159	1.000	7.94 × 10^−2^	1.66 × 10^−3^
313.15	1.679	2.187	1.000	9.33 × 10^−2^	1.78 × 10^−3^

**Table 8:** Model parameters, *R*^2^, RAD, and RMSD values of the MRS model by fitting the solubility data.

*T*/K	MRS Model							
	*β*_1_	*β*_2_	*β*_3_	*β*_4_	*β*_5_	*R* ^2^	RAD	RMSD
293.15	−2.728	−12.141	−0.011	0.006	9.402	1.000	7.92 × 10^−2^	1.71 × 10^−3^
298.15	−2.643	−11.900	−0.011	0.007	9.178	1.000	7.80 × 10^−2^	2.27 × 10^−3^
303.15	−2.576	−11.697	−0.011	0.007	9.069	1.000	7.57 × 10^−2^	2.04 × 10^−3^
308.15	−2.533	−11.514	−0.011	0.008	9.052	1.000	9.21 × 10^−2^	2.56 × 10^−3^
313.15	−2.456	−11.307	−0.011	0.008	8.909	0.999	1.16 × 10^−1^	3.09 × 10^−3^

**Table 9:** ln*x* values of MHY498 calculated by Yalkowsky-Roseman model in DEGME (1) + water (2) mixtures at 293.15 to 313.15 K.

*w*_1_	ln*x*				
	*T* = 293.15 K	*T* = 298.15 K	*T* = 303.15 K	*T* = 308.15 K	*T* = 313.15 K
0.0	−12.29	−12.09	−11.88	−11.70	−11.50
0.1	−11.30	−11.12	−10.91	−10.74	−10.56
0.2	−10.32	−10.14	−9.95	−9.79	−9.61
0.3	−9.33	−9.16	−8.99	−8.83	−8.67
0.4	−8.35	−8.19	−8.02	−7.88	−7.72
0.5	−7.36	−7.21	−7.06	−6.92	−6.78
0.6	−6.37	−6.24	−6.10	−5.97	−5.83
0.7	−5.39	−5.26	−5.13	−5.02	−4.89
0.8	−4.40	−4.28	−4.17	−4.06	−3.95
0.9	−3.42	−3.31	−3.21	−3.11	−3.00
1.0	−2.43	−2.33	−2.24	−2.15	−2.06
RAD	1.41	1.40	1.39	1.38	1.37
RMSD	2.19 × 10^−2^	2.36 × 10^−2^	2.51 × 10^−2^	2.67 × 10^−2^	2.86 × 10^−2^

**Table 10:** Thermodynamic parameters of MHY498 dissolution in the monosolvents at 302.99 K.

Solvent	∆*H*° (kJ∙mol^−1^)	∆*G*° (kJ∙mol^−1^)	∆*S*° (J∙mol^−1^K^−1^)	*T*∆*S*° (kJ∙mol^−1^)	*%ζ_H_*	*%ζ_TS_*
Acetone	16.08	8.02	26.63	8.06	66.60	33.40
Acetonitrile	101.39	18.02	275.16	83.37	54.88	45.12
1-Butanol	14.80	10.78	13.29	4.03	78.62	21.38
DEGME	14.17	5.65	28.10	8.51	62.46	37.54
DMA	13.56	5.20	27.60	8.36	61.86	38.14
DMF	9.68	4.98	15.52	4.70	67.30	32.70
DMSO	19.74	5.43	47.22	14.31	57.98	42.02
Ethanol	9.47	9.34	0.40	0.12	98.74	1.26
Ethyl acetate	43.29	14.88	93.77	28.41	60.37	39.63
Methanol	11.09	10.88	0.71	0.22	98.90	1.91
NMP	15.20	5.96	30.51	9.24	62.19	37.81
1-Propanol	10.65	10.55	0.31	0.09	99.12	0.88
2-Propanol	13.08	9.71	11.14	3.37	79.50	20.50
THF	48.09	6.24	138.12	41.85	53.47	46.53
Water	30.08	29.95	0.40	0.12	99.60	0.40

**Table 11:** Thermodynamic parameters of MHY498 dissolution in DEGME (1) + water (2) mixtures at 302.99 K.

*w*_1_	∆*H*° (kJ∙mol^−1^)	∆*G*° (kJ∙mol^−1^)	∆*S*° (J∙mol^−1^∙K^−1^)	*T*∆*S°* (kJ∙mol^−1^)	*%ξ_H_*	*%ξ_TS_*
0.0	30.08	29.95	0.40	0.12	99.60	0.40
0.1	24.92	24.73	0.60	0.18	99.27	0.73
0.2	23.55	20.95	8.61	2.61	90.03	9.97
0.3	24.86	17.65	23.78	7.21	77.53	22.47
0.4	23.03	14.37	28.57	8.66	72.68	27.32
0.5	12.68	12.32	1.18	0.36	97.25	2.75
0.6	10.72	10.37	1.16	0.35	96.84	3.16
0.7	14.48	8.81	18.71	5.67	71.86	28.14
0.8	12.32	7.58	15.67	4.75	72.19	27.81
0.9	12.30	6.55	18.99	5.75	68.13	31.87
1.0	14.17	5.65	28.10	8.51	62.46	37.54

## Data Availability

Data are contained within the article.

## Supplementary Materials

The following supporting information can be downloaded at: https://www.mdpi.com/article/10.3390/pharmaceutics18010127/s1, Table S1: Properties of MHY498 and the solvents used in the present study; Table S2: Preparation of MHY498 nanosuspensions using different solvents by antisolvent precipitation; Table S3: Calculation of molar volume and Hildebrand solubility parameter of MHY498 using Fedor’s technique; Table S4: Solvatochromatic parameters (α, β, and π*) and Hildebrand solubility parameter (δ_H_) for the mono-solvents; Table S5: Parameters value of KAT-LSER model for solubility data of MHY498 in monosolvents; Table S6: ANOVA table obtained from Box–Behnken design; Table S7: Long-term stability of the optimized MHY498 nanosuspension (particle size 28.1 nm).
