# Supplementary material for: MHY498 Nanosuspensions for Improved Topical Drug Delivery: Understanding of Its Solubility Behavior in DEGME + Water Mixtures and Preparation of Nanosuspension Using Box–Behnken Design
Source: Pharmaceutics. 2026 Jan 20;18(1):127. doi: 10.3390/pharmaceutics18010127 (PMC12845458; doi:10.3390/pharmaceutics18010127)
Supplement: Supplementary file 1 [file pharmaceutics-18-00127-s001.zip › pharmaceutics-4097423-supplementary.pdf]

Supplementary material

MHY498 Nanosuspensions for Improved Topical Drug Delivery: Understanding of Its Solubility Behavior in DEGME + Water Mixtures and Preparation of Nanosuspension Using Box–Behnken Design

Eun-Sol Ha [1,†], Ha Nim Lee [1,†], Seon-Kwang Lee [1], Ji-Su Jeong [1], Jeong-Soo Kim [2], Hyung Ryong Moon [1], In-hwan Baek [3], HeejunPark [4,*], Min-Soo Kim [1,*]

[1] *College of Pharmacy and Research Institute for Drug Development, Pusan National University, 63 Busandaehak-ro, Geumjeong-gu, Busan 46241, Republic of Korea*

[2] *Dong-A ST Co., Ltd., Giheung-gu, Yongin 17073, Republic of Korea*

[3] *College of Pharmacy, Kyungsung University, 309, Suyeong-ro, Nam-gu, Busan 48434, Republic of Korea*

[4] *College of Pharmacy, Duksung Women's University, 33, Samyangro 144-gil, Dobong-gu, Seoul 01369, Republic of Korea*

[*] Author to whom correspondence should be addressed.; *E-mail address*: minsookim@pusan.ac.kr (M.-S. Kim); heejunpark@duksung.ac.kr (H.Park)

[†] These authors contributed equally to this work.

Table S1. Properties of MHY498 and the solvents used in the present study.

Materials	Molecular formula	Molar mass ($g \cdot mol^{-1}$)	Purity mass fraction[a]
MHY498	$C_{10}H_7NO_4S$	237.23	0.999
Acetone	C_3H_6O	58.08	0.995
Acetonitrile	C_2H_3N	41.05	>0.999
1-Butanol	C_4H_9OH	74.12	>0.995
DEGME	$C_6H_{14}O_3$	134.17	>0.999
DMA	C_4H_9NO	87.12	>0.999
DMF	C_3H_7NO	73.09	>0.999
DMSO	C_2H_6OS	78.13	>0.999
Ethanol	C_2H_5OH	46.07	0.999
Ethyl acetate	$C_4H_8O_2$	88.11	0.999
Methanol	CH_3OH	32.04	0.999
NMP	C_5H_9NO	99.13	>0.997
1-Propanol	$CH_3CH_2CH_2OH$	60.10	>0.995
2-Propanol	$(CH_3)_2CHOH$	60.10	>0.997
THF	C_4H_8O	72.11	0.998
Water	H_2O	18.01	>0.999

[a] The mass fraction purity values were provided by the suppliers.

Table S2. Preparation of MHY498 nanosuspensions using different solvents by antisolvent precipitation.

Solvent	Particle size (nm) [b]	PDI
DMF	2065.6 ± 198.5	0.557
DMA	3798.6 ± 140.6	1.444
DMSO	3303.0 ± 452.9	1.198
DEGME	65.8 ± 11.1	0.250

[a] All formulations were prepared under identical antisolvent precipitation conditions (drug mole fraction: 0.042; PVP K30 concentration: 2 % w/w; solvent-to-antisolvent ratio: 0.15; stirring speed: 500 rpm; temperature: 298.15 K).

[b] Particle sizes represent mean ± SD (n = 3).

Table S3. Calculation of molar volume and Hildebrand solubility parameter of MHY498 using Fedor's technique.

Group	Group number	Δ_{ei} (cal·mol^{-1})	Δ_{vi} (cm^3·mol^{-1})
-CH=	1	1030	13.5
C=	1	1030	-5.5
Phenylene (m)	1	7630	52.4
Ring closure 5 or more atoms	1	250	16
>C=O	1	4150	10.8
CONH	1	8000	9.5
-OH	2	7120	10
-S-	1	3380	12
Total		39710	128.7
Solubility parameter	$(39710/128.7)^{1/2} = 17.57\ (\text{cal·cm}^{-3})^{1/2} = 35.83\ \text{MPa}^{1/2}$		

Table S4. Solvatochromatic parameters (α, β, and π^*) and Hildebrand solubility parameter (δ_H) for the mono-solvents.

Solvent	α	β	π^*	δ_H ($MPa^{1/2}$)
Acetone	0.08	0.43	0.71	19.95
Acetonitrile	0.19	0.40	0.75	24.40
1-Butanol	0.84	0.84	0.47	23.35
DMA		0.76	0.88	22.77
DMF		0.69	0.88	23.95
DMSO		0.76	1.00	26.75
Ethanol	0.86	0.75	0.54	26.13
Ethyl acetate		0.45	0.55	18.48
Methanol	0.98	0.66	0.60	29.61
NMP		0.77	0.92	22.96
1-Propanol	0.84	0.90	0.52	24.45
2-Propanol	0.76	0.84	0.48	23.58
THF		0.54	0.51	19.46
Water	1.17	0.47	1.09	47.82

Taken from Refs. [37−40]

Table S5. Parameters value of KAT-LSER model for solubility data of MHY498 in monosolvents.

C_0	C_1	C_2	C_3	C_4	Squared correlation coefficient (R^2)	*F*-statistic values
-8.36 (2.39)	-0.98 (2.83)	7.60 (3.35)	2.86 (5.13)	-7.60 (5.85)	0.82	10.45
-7.31 (2.32)	-4.48 (0.91)	10.70 (2.44)	-3.33 (1.95)		0.79	12.51
-7.60 (1.90)	-2.42 (1.15)	8.77 (2.53)		-4.57 (2.08)	0.82	14.86
-7.00 (2.75)	3.67 (2.32)		10.11 (4.77)	-17.05 (4.90)	0.72	8.63
**-8.66 (2.13)**		**6.76 (2.21)**	**4.48 (2.06)**	**-9.54 (1.74)**	**0.82**	**15.24**
-10.20 (1.72)	-4.15 (0.97)	11.31 (2.61)			0.73	14.75
0.30 (2.53)	-3.58 (1.45)		-4.60 (3.15)		0.38	3.42
-1.41 (0.90)	-0.49 (1.42)			-7.87 (2.62)	0.60	8.13
-8.80 (4.05)		8.03 (4.18)	-1.31 (3.36)		0.28	2.17
-5.81 (1.94)		6.18 (2.53)		-7.59 (1.73)	0.74	15.28
-3.45 (1.70)			3.72 (2.71)	-10.10 (2.30)	0.65	10.30
-3.17 (0.89)	-3.05 (1.47)				0.26	4.31
-9.95 (2.69)		8.37 (3.94)			0.27	4.50
-2.56 (2.68)			-2.63 (3.64)		0.04	0.52
-1.42 (0.87)				-8.42 (2.02)	0.59	17.42

Table S6. ANOVA table obtained from Box-Behnken design.

Source	Sum of Squared	df	Mean Square	*F*-value	*p*-value	
Model	11154.30	9	1239.37	29.78	0.0008	*significant*
X_1-Drug concentration	780.13	1	780.13	18.74	0.0075	
X_2- PVP K30 concentration	7925.40	1	7925.40	190.41	< 0.0001	
X_3-Solvent to antisolvent ratio	420.50	1	420.50	10.10	0.0246	
X_1X_2	1249.62	1	1249.62	30.02	0.0028	
X_1X_3	63.20	1	63.20	1.52	0.2726	
X_2X_3	167.70	1	167.70	4.03	0.1010	
X_1^2	390.13	1	390.13	9.37	0.0281	
X_2^2	195.64	1	195.64	4.70	0.0823	
X_3^2	1.70	1	1.70	0.0409	0.8477	
Residual	208.11	5	41.62			
Lack of Fit	200.31	3	66.77	17.11	0.0557	*not significant*
Pure Error	7.81	2	3.90			
Cor Total	11362.41	14				
Standard deviation	6.45					
R^2	0.9817					
Adjusted R^2	0.9487					
Predicted R^2	0.7164					

Table S7. Long-term stability of the optimized MHY498 nanosuspension (particle size 28.1 nm).

Months	Particle size (nm) [b]	Polydispersity index
Initial	28.1 ± 0.5	0.162
1 month	30.5 ± 0.8	0.178
3 months	29.7 ± 0.7	0.165
6 months	31.1 ± 0.9	0.189

[a] All formulations were stored under identical storage conditions at long-term condition (298.15 K, 60% RH) and evaluated at predetermined time points.

[b] Particle sizes represent mean ± SD (n = 3).
